# Phase Evolution and Dynamic Response of Tungsten–Zirconium Alloys: Insights into W_2_Zr Inhibition via W/Zr Ratio Tailoring

**DOI:** 10.3390/ma19102097

**Published:** 2026-05-16

**Authors:** Hongtai Yang, Yu Xuan, Kai Liu, Liang Ren, Kongxun Zhao, Xiang Li, Wei Huang, Guitao Liu

**Affiliations:** 1 China Academy of Ordnance Science Ningbo Branch, Ningbo 315103, China; hongtai9905@163.com (H.Y.);; 2 National Elite Institute of Engineering, Northwestern Polytechnical University, Xi’an 710072, China; 3 School of Mechanical Engineering, Northwestern Polytechnical University, Xi’an 710072, China

**Keywords:** tungsten–zirconium alloys, energetic structural materials, W_2_Zr formation, microstructure regulation

## Abstract

**Highlights:**

- This study is the first proposed alloy design strategy for suppressing W_2_Zr formation through a low-temperature eutectic reaction.
- Nanoscale refinement of the W_2_Zr phase was achieved by introducing the Ti and Ni elements, resulting in a novel WZrTiNi alloy with a low W_2_Zr volume fraction.
- A synergistic improvement in dynamic compressive strength and energy-release efficiency is achieved via a tailored microstructure of the W-Zr alloys.

**Abstract:**

The formation of coarse brittle W_2_Zr phases severely limits the dynamic mechanical performance of energetic W-Zr structural materials. In this work, Ti and Ni were introduced into the W-Zr system to modify the phase evolution during sintering, and three alloys with different Zr atomic concentrations, W*_x_*Zr_85−*x*_Ti_7.5_Ni_7.5_ (*x* = 45, 55, and 65), were prepared by vacuum sintering. Microstructural characterization showed that the W_45_Zr_40_Ti_7.5_Ni_7.5_ alloy contained abundant coarse micron-sized W_2_Zr particles, whereas both the W_55_Zr_30_Ti_7.5_Ni_7.5_ and W_65_Zr_20_Ti_7.5_Ni_7.5_ alloys exhibited a lower fraction of W_2_Zr together with a much finer characteristic size. In particular, decreasing the Zr content reduced the characteristic size of W_2_Zr from several micrometers to below 200 nm. Interrupted sintering and thermal analyses suggest that the preferential formation of a Zr(Ti) solid solution and a Zr-Ti-Ni-rich ternary phase at lower temperatures reduces the local availability of free Zr for reaction with W, thereby suppressing the nucleation and growth of W_2_Zr. Correspondingly, the dynamic compressive strength increased from 1054 MPa for W_45_Zr_40_Ti_7.5_Ni_7.5_ to 1720 MPa for W_65_Zr_20_Ti_7.5_Ni_7.5_. In addition, the W_65_Zr_20_Ti_7.5_Ni_7.5_ alloy maintained pronounced impact-induced reaction behavior despite its lower Zr content. These results indicate that tailoring the W/Zr ratio in the Ti/Ni-containing W-Zr system provides a feasible route to regulate W_2_Zr formation and improve the compressive response under dynamic loading.

## 1. Introduction

As a representative system of energetic structural materials (ESMs), W-Zr alloys constitute a class of novel, high-efficiency damage-enhancing materials that combine structural load-bearing capacity with energy-release capability [1,2,3]. Under ambient conditions, ESMs exhibit excellent mechanical stability and structural integrity, thereby meeting the load-bearing requirements of conventional projectile components. Under extreme loading conditions such as impact or detonation, the material fragments and initiates a vigorous exothermic chemical reaction, generating multiple secondary damage effects including high overpressure, elevated temperatures, and incendiary action, thereby significantly enhancing its lethality [4,5,6]. This “structure-function integration” characteristic positions ESMs as a key research direction for overcoming the performance limitations of conventional inert damage materials.

Since the energy release of W-Zr alloys primarily relies on oxidation reactions that occur after fragmentation upon impact with a target plate [7,8,9], these alloys should not exhibit excessive ductility, as it would impede sufficient fragmentation. However, excessive brittleness can cause premature fragmentation under detonation loading, leading to premature consumption of their chemical energy during the initial launch phase or in flight. Therefore, from the perspectives of service conditions and reaction mechanisms, an ideal energetic fragment material should be a brittle material with a high elastic deformation limit [10,11,12]. However, the elastic deformation limit of current W-Zr alloys is relatively low, typically below 1000 MPa [13,14], making them highly susceptible to failure under high-rate detonation loading. This issue is particularly critical in the context of the ongoing trend toward higher-energy energetic materials, where W-Zr alloys are increasingly unable to withstand the more demanding initiation velocities [15,16].

The primary factor limiting the elastic deformation limit of W-Zr alloys is the brittle W_2_Zr phase formed during processing [17]. This phase typically exhibits an irregular polygonal morphology, with grain sizes ranging from several to tens of micrometers. Under dynamic impact loading, severe stress concentration tends to occur at the sharp corners of the W_2_Zr particles or at the weak interfaces between these particles and the surrounding matrix [18,19,20]. Consequently, even when the overall applied stress remains low, the local stress may already reach either the fracture strength of the brittle W_2_Zr phase or the interfacial bond strength between the brittle phase and the matrix, leading to premature crack initiation and rapid propagation. Macroscopically, this results in brittle fracture at low stress levels and an extremely short elastic deformation stage. According to the W-Zr binary equilibrium phase diagram, the formation of W_2_Zr is thermodynamically unavoidable, regardless of the W-to-Zr compositional ratio [21]. Given the difficulty in altering the phase constitution, tailoring the morphology and spatial distribution of the W_2_Zr phase represents a practical and viable technological route to enhance the elastic deformation limit of W-Zr alloys.

The morphology and distribution of phases are fundamentally governed by their nucleation and growth, which in turn are controlled by temperature and concentration. Since both W and Zr are refractory elements with high melting points, the chemical reaction between them is kinetically limited by slow diffusion and thus predominantly occurs above 900 °C. Higher temperatures further promote the formation and growth of the W_2_Zr phase [22,23,24]. Consequently, lowering the sintering temperature represents a viable strategy to suppress W_2_Zr formation. In addition, from the perspective of concentration effects, reducing the local Zr atomic concentration in the vicinity of W can similarly inhibit the nucleation and growth of W_2_Zr. However, since Zr serves as the primary source of energy release in energetic fragments, this reduction cannot be achieved simply by decreasing the overall Zr content. Instead, it requires the introduction of additional alloying elements to modulate the local chemical environment and suppress the W-Zr reaction through competitive alloying interactions. Thermodynamic calculations based on ternary phase diagrams indicate that Zr undergoes eutectic reactions with Ti and Ni at relatively low temperatures—below 800 °C [25]. Therefore, incorporating Ti and Ni as binder-phase elements into W-Zr alloys enables these low-temperature eutectic reactions with Zr, which not only facilitate densification during sintering at reduced temperatures but also partially deplete the local Zr concentration around W particles, thereby effectively suppressing the nucleation and growth of W_2_Zr.

Based on this rationale, this study proposes a novel W-Zr alloy system, in which Ti and Ni are introduced as alloying modifiers. Three compositions with varying Zr atomic concentrations—W*_x_*Zr_85−*x*_Ti_7.5_Ni_7.5_ (*x* = 45, 55, and 65)—were designed to systematically investigate the role of Zr content. The low-melting eutectic reactions among Ti, Ni, and Zr are leveraged to lower the sintering temperature while simultaneously suppressing the nucleation and growth of brittle intermetallic phases. This work focuses on elucidating the effects of Zr atomic concentration on microstructural evolution, phase constitution, and the elastic deformation limit of the alloy, thereby providing a theoretical foundation for the development of high-performance energetic structural materials and the optimization of their processing parameters.

## 2. Materials and Methods

**Figure 1:**
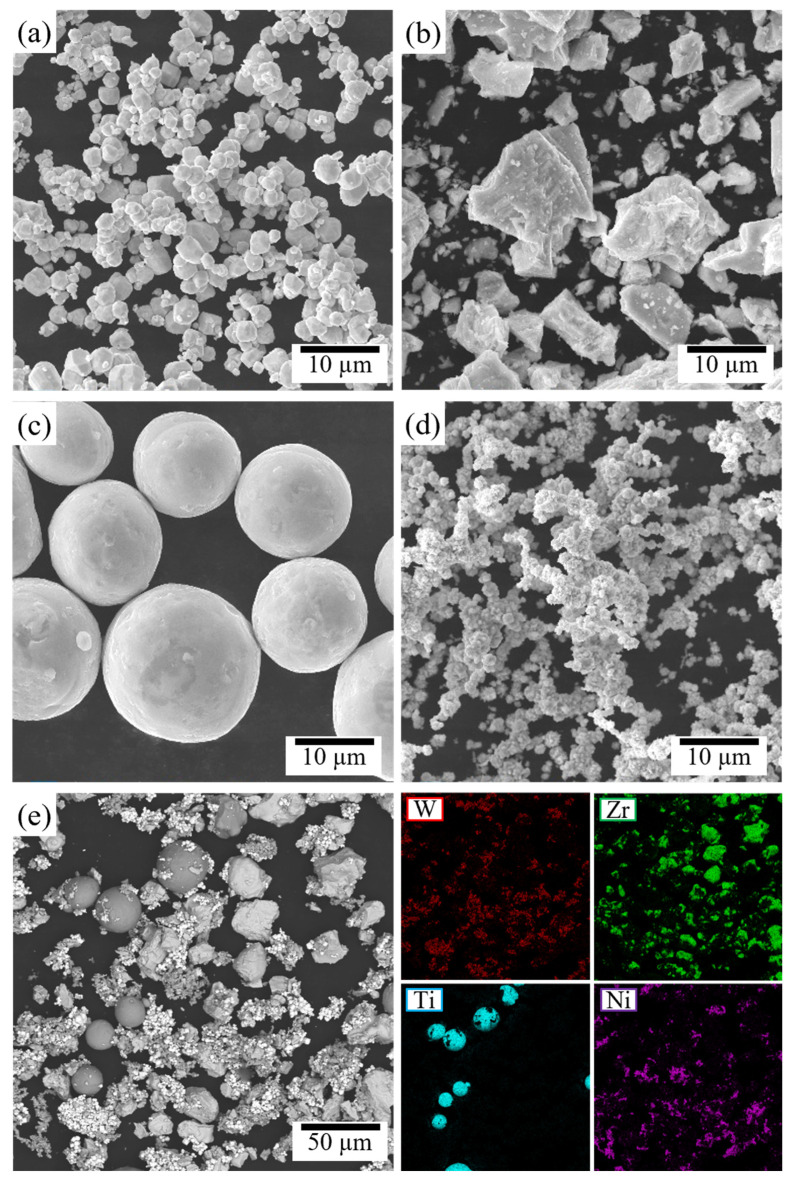
Morphology images of the original powders: (**a**) W; (**b**) Zr; (**c**) Ti; (**d**) Ni; and (**e**) granulated powder and its elemental distribution.

This study designed three quaternary W*_x_*Zr_85−*x*_Ti_7.5_Ni_7.5_ alloy systems with varying Zr atomic concentrations, and the corresponding compositional details are listed in Table 1. The raw materials consisted of W powder (particle size: 3–4 μm, 99.9% purity), Zr powder (5–10 μm, 99.9% purity), Ti powder (9–11 μm, 99.9% purity) and Ni powder (3–4 μm, 99.9% purity). The raw metal powders were subjected to ball milling for 6 h at a rotational speed of 120 rpm and a ball-to-powder mass ratio of 4:1 to achieve homogeneous mixing. Subsequently, an aqueous polyvinyl alcohol (PVA) binder solution, accounting for 6 wt% of the total powder mass, was added to the blended powder for granulation. This process yielded agglomerated granules with good flowability and a uniform particle size distribution, which were used as feedstock for subsequent sintering. The morphologies of the as-received raw powders are presented in Figure 1. The EDS results of the granulated powder are shown in Figure 1e. The granulated powders were uniaxially pressed into 10 × 10 mm green compacts using an automatic hydraulic press under a pressure of 200 MPa.

The sintering process involved cold pressing followed by high-vacuum sintering. The heating rate was maintained at 10 °C/min throughout the process. Two isothermal holds were applied during heating to ensure complete removal of the polyvinyl alcohol binder. The first hold was at 450 °C, and the second was set at 650 °C. These steps enabled staged thermal decomposition and full elimination of the binder. After that, the temperature was raised to 1050 °C and held for 2 h. Finally the samples were furnace-cooled to room temperature. After sintering the alloy samples achieved a relative density exceeding 99%. This corresponds to nearly full densification.

The phase constitution of the alloys was characterized by X-ray diffraction (XRD) (Bruker Corporation, Billerica, MA, USA), scanning electron microscopy (SEM) (Thermo Fisher Scientific, Hillsboro, OR, USA) and Transmission Electron Microscopy (TEM) (Thermo Fisher Scientific Inc., Eindhoven, The Netherlands), while elemental distributions were analyzed by energy-dispersive X-ray spectroscopy (EDS) (Bruker Nano GmbH, Berlin, Germany). To clarify the phase evolution mechanism, the alloying reactions during the sintering of the quaternary system were investigated by Differential Scanning Calorimetry (DSC), and further microstructural analysis of the W_55_Zr_30_Ti_7.5_Ni_7.5_ alloy was conducted to confirm the mechanism responsible for W_2_Zr suppression. The specimens were cut into cylindrical samples with dimensions of Φ8 × 8 mm and Φ4 × 2 mm using a precision diamond wire saw (Diamond WireTec GmbH & Co. KG (DWT), Weinheim, Germany). Subsequently, quasi-static compression tests were performed on Φ8 × 8 mm cylinders at a strain rate of 10^−3^ s^−1^ using a universal testing machine (Instron Corporation, Norwood, MA, USA). Dynamic tests were conducted using a split Hopkinson pressure bar (SHPB) (Instron Corporation, Norwood, MA, USA) on Φ4 × 2 mm specimens to capture the high-strain-rate response. The equipment used in the experiments is listed in Table 2.

## 3. Results and Discussion

### 3.1. Microstructural Evolution Analysis

**Figure 2:**
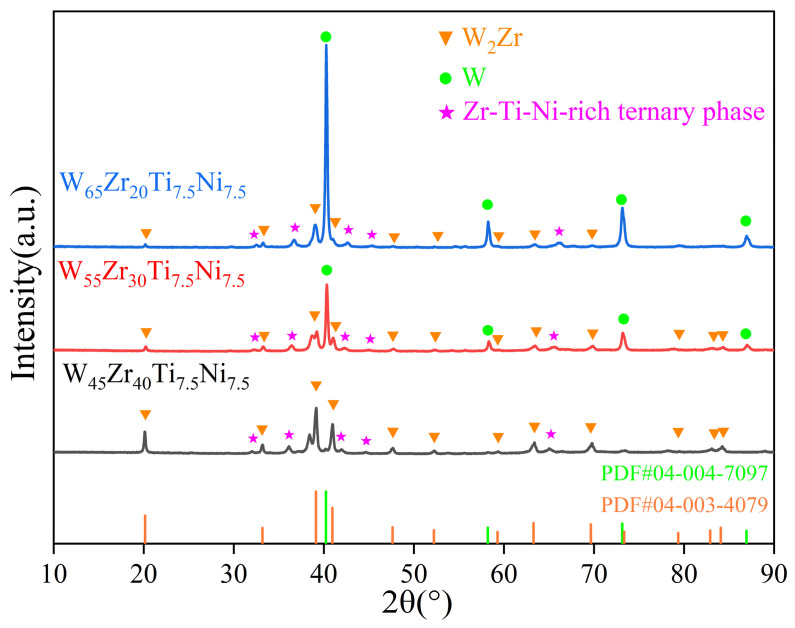
XRD patterns of W*_x_*Zr_85−*x*_Ti_7.5_Ni_7.5_ alloys.

Figure 2 presents the XRD patterns of the W*_x_*Zr_85−_*_x_*Ti_7.5_Ni_7.5_ alloys with varying Zr contents. Phase identification reveals a systematic evolution in phase composition: the W_45_Zr_40_Ti_7.5_Ni_7.5_ alloy is dominated by the W_2_Zr intermetallic phase with negligible elemental W. Starting from W_55_Zr_30_Ti_7.5_Ni_7.5_, the diffraction peaks of elemental W become clearly detectable, and in the W_65_Zr_20_Ti_7.5_Ni_7.5_ alloy, elemental W becomes the predominant phase, accompanied by reduced W_2_Zr content. All compositions exhibit a diffraction feature that does not match any entry in the ICDD PDF database and are tentatively assigned to a Zr-Ti-Ni-rich ternary phase. This phase will be further verified through subsequent characterization. The crystallographic information of the identified phases is referenced from the standard PDF database: elemental tungsten (W) has a body-centered cubic (BCC) structure with the space group *Im3m* (No. 229), a standard lattice parameter of *a* = 3.167 Å, and a density of 19.222 g/cm^3^ (PDF#04-004-7097). The W_2_Zr phase exhibits a face-centered cubic (FCC) structure with the space group *Fd3m* (No. 227), a standard lattice parameter of *a* = 7.63 Å, and a density of 13.725 g/cm^3^ (PDF#04-003-4079). This indicates that under the employed sintering conditions, the system stabilizes into a thermodynamically stable multiphase microstructure. In the W_45_Zr_40_Ti_7.5_Ni_7.5_ alloy, the diffraction peak intensity of W_2_Zr is significantly stronger than that in other compositions, while the diffraction peak of elemental W has nearly disappeared. This suggests that the high Zr content is sufficient to consume most of the W present, leading to predominant formation of the W_2_Zr phase. With decreasing Zr content, the relative intensity of the W_2_Zr diffraction peaks gradually weakens, while that of elemental W correspondingly increases. This trend reflects a progressive reduction in the W_2_Zr volume fraction, alongside an increasing amount of unreacted elemental W. These results indicate that the incorporation of Ti and Ni as binder-phase elements, combined with tailored Zr content, partially suppresses the excessive precipitation of the brittle W_2_Zr phase [26].

**Figure 3:**
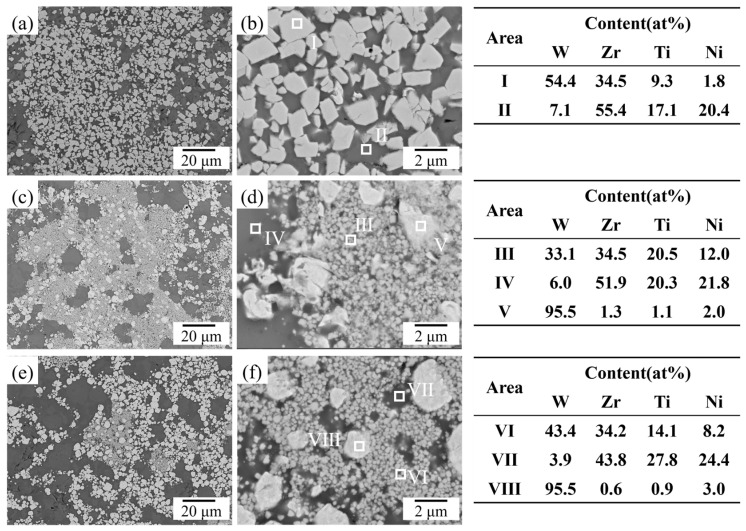
BSE images and EDS results of the W*_x_*Zr_85−*x*_Ti_7.5_Ni_7.5_ alloys: (**a**,**b**) W_45_Zr_40_Ti_7.5_Ni_7.5_ alloy; (**c**,**d**) W_55_Zr_30_Ti_7.5_Ni_7.5_ alloy; and (**e**,**f**) W_65_Zr_20_Ti_7.5_Ni_7.5_ alloy.

**Figure 4:**
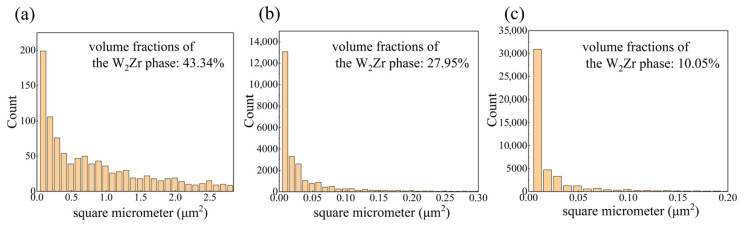
Variation in the volume fraction of the W_2_Zr phase: (**a**) W_45_Zr_40_Ti_7.5_Ni_7.5_ alloy; (**b**) W_55_Zr_30_Ti_7.5_Ni_7.5_ alloy; and (**c**) W_65_Zr_20_Ti_7.5_Ni_7.5_ alloy.

Subsequently, the cross-sectional microstructure of the sintered samples was examined by SEM. Figure 3a shows the SEM-BSE image of the W_45_Zr_40_Ti_7.5_Ni_7.5_ alloy, and Figure 3b provides a detailed view of the local region. As shown, the W_45_Zr_40_Ti_7.5_Ni_7.5_ alloy is primarily composed of two phases: the light-gray Phase I with sizes ranging from 2~5 μm and the dark matrix Phase II. According to the EDS date, the light-gray Phase I contains 54.4 at% W and 34.5 at% Zr. Combined with the XRD results, this phase is identified as W_2_Zr. As shown in Figure 4, the volume fraction of Phase I was calculated to be 43.34%. The dark Phase II is composed of Zr, Ti and Ni and is identified as a Zr-Ti-Ni-rich ternary phase. In the case of the W_55_Zr_30_Ti_7.5_Ni_7.5_ alloy, a significant change in microstructure is observed. As shown in Figure 3c,d, in addition to the bright W phase (Phase V), a large number of light-gray nanoparticles are observed within the alloy. EDS analysis indicates that the light-gray Phase III contains 33.1 at% W and 34.5 at% Zr, suggesting that it is a W-Zr intermetallic compound. The volume fraction of Phase III is 27.95%. The dark Phase IV exhibits an elemental composition nearly identical to that of Phase II and is assigned to the Zr-Ti-Ni-rich ternary phase. The microstructure of the W_65_Zr_20_Ti_7.5_Ni_7.5_ alloy (Figure 3e) is similar to that of the W_55_Zr_30_Ti_7.5_Ni_7.5_ alloy, comprising the light-gray Nanophase VI, the dark Phase VII and the granular gray Phase VIII. The volume fraction of Phase VI further decreased to 10.05%. It should be noted that with a further decrease in Zr content, the volume fraction of the Zr-Ti-Ni-rich ternary phase increases notably, while that of the W-Zr intermetallic compound is significantly reduced. This suggests a competitive relationship between the formation of the Zr-Ti-Ni-rich ternary phase and the W-Zr intermetallic compound.

**Figure 5:**
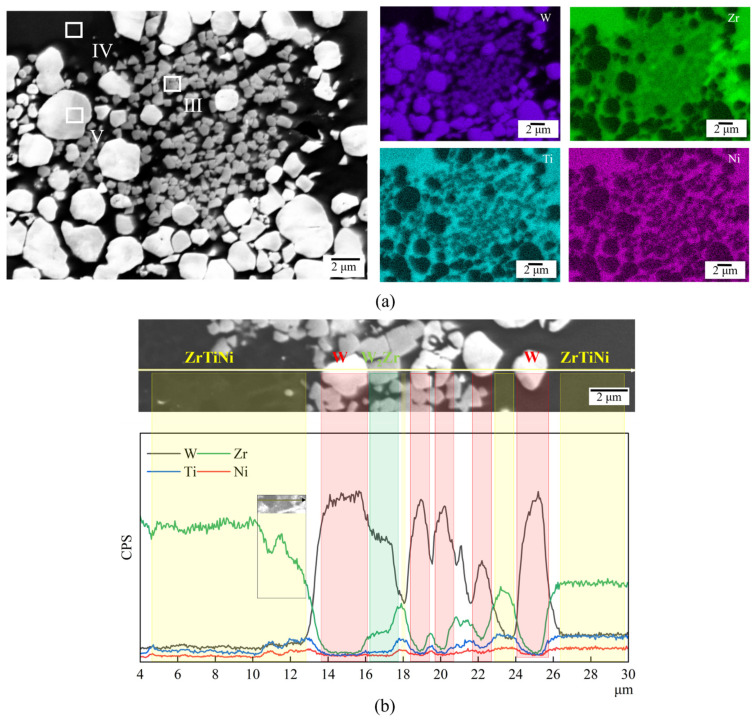
EDS results of the W_55_Zr_30_Ti_7.5_Ni_7.5_ alloy: (**a**) elemental mapping and (**b**) line-scan analysis.

EDS mapping and line-scan analyses were conducted on the W_55_Zr_30_Ti_7.5_Ni_7.5_ alloy sample, with the results shown in Figure 5. As illustrated, the four metallic elements (W, Zr, Ti, and Ni) exhibit a distinct and systematic spatial distribution within the microstructure. W is predominantly enriched in the irregularly shaped light-gray Phase III and the granular gray Phase V. Zr is mainly distributed in Phases III and IV, whereas Ti and Ni are primarily confined to the dark contrast Phase IV.

**Figure 6:**
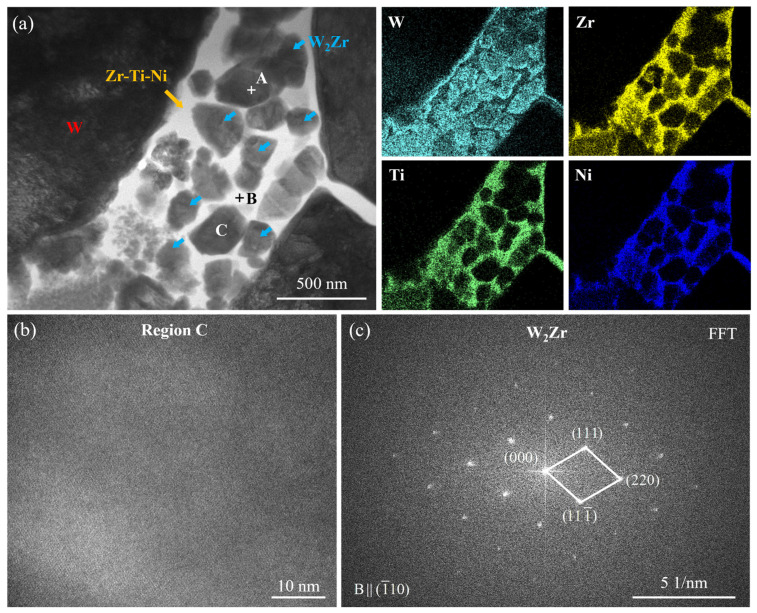
TEM observation and analysis of the W_55_Zr_30_Ti_7.5_Ni_7.5_ alloy: (**a**) bright-field TEM image and elemental mapping; (**b**) high-resolution TEM image of Region C; and (**c**) corresponding FFT analysis result of the HRTEM image in Region C.

To further determine the composition of the W-Zr intermetallic compound (Phase III), detailed transmission electron microscopy analysis was conducted on the W_55_Zr_30_Ti_7.5_Ni_7.5_ alloy sample, with results presented in Figure 6. Bright-field TEM imaging revealed fine, nanoscale precipitates dispersed within the microstructure, with a representative particle at point A selected for detailed investigation. As shown in Table 3, energy-dispersive X-ray spectroscopy performed at this location yielded an atomic W-to-Zr ratio of approximately 2:1, which is in excellent agreement with the stoichiometry expected for the W_2_Zr phase. Elemental mapping further clarified the spatial distribution of constituents: the nanoparticles are distinctly enriched in W and Zr, confirming their identity as a W-Zr-based intermetallic, while Ti and Ni are absent from these particles. The FFT diffraction spots corresponding to the high-resolution image are consistent with the Laves structure of W_2_Zr, thereby unambiguously identifying this phase. The electron diffraction patterns of the Zr-Ti-Ni-rich ternary phase are diffuse or unindexable, likely due to severe lattice distortion caused by mutual atomic substitution among Zr, Ti, and Ni. Although its precise crystal structure remains undetermined and no matching phase can be found in existing standard databases, EDS elemental mapping consistently reveals the coexistence of Zr, Ti, and Ni in these regions, strongly supporting the assignment of this phase as a Zr-Ti-Ni-rich ternary phase.

Overall, the microstructural results show that decreasing the Zr content in the present Ti/Ni-containing system reduces both the fraction and characteristic size of W_2_Zr while increasing the amount of W retained and the Zr-Ti-Ni-rich ternary phase. These changes are expected to alleviate the severe local stress concentration associated with coarse brittle intermetallics and are therefore relevant to the improved compressive response discussed below.

### 3.2. Suppression Mechanism of W_2_Zr Intermetallic

**Figure 7:**
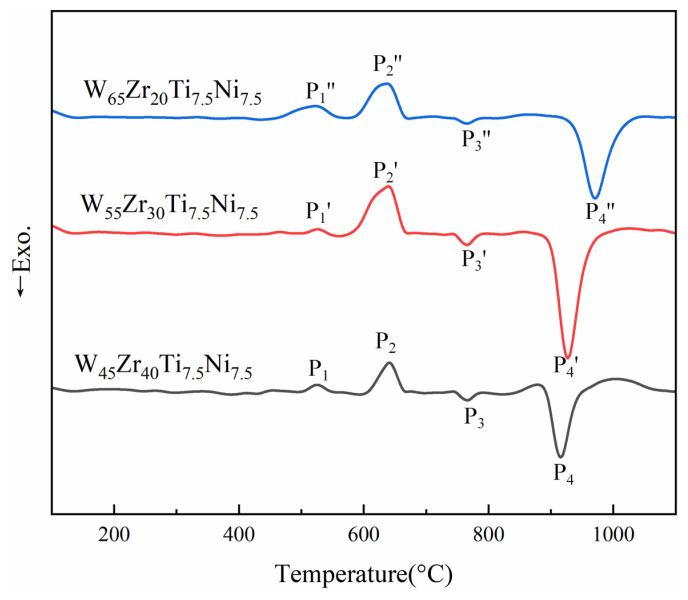
DSC curves of the W*_x_*Zr_85−*x*_Ti_7.5_Ni_7.5_ mixed powders.

To explore the microstructural evolution mechanism in W*_x_*Zr_85−*x*_Ti_7.5_Ni_7.5_ alloys, DSC tests and microstructural analyses were performed on samples sintered at different temperatures. Figure 7 presents the DSC curves of W*_x_*Zr_85−*x*_Ti_7.5_Ni_7.5_ mixed powders, showing that the types of thermal reactions are essentially identical across all compositions. Taking the W_45_Zr_40_Ti_7.5_Ni_7.5_ alloy as example, a weak endothermic peak appears upon heating to 525 °C. Based on the Zr-Ti binary phase diagram [27], this peak is attributed to the formation of an HCP Zr(Ti) solid solution. Subsequently, a pronounced endothermic peak (P_2_) emerges at approximately 645 °C, and it is primarily associated with the decomposition of the granulation binder. This assignment is corroborated by the observed changes in vacuum pressure during sintering within the 600–650 °C temperature range. Following this, an exothermic peak (P3) appears near 760 °C. According to the Ti-Ni binary phase diagram and corroborating literature reports [28], this event corresponds to an interfacial reaction between Ni and the pre-formed Zr(Ti) solid solution, leading to the nucleation and growth of a complex Zr-Ti-Ni-based intermetallic compound. This low-temperature exothermic reaction is particularly significant, as it indicates enhanced atomic mobility and reactivity among the Ti, Ni and Zr even well below the melting points of the constituent metals, thereby facilitating early-stage densification and microstructural homogenization. The assignment of the above reaction peaks is not only based on the reported literature but also corroborated by XRD patterns obtained after sintering at different holding times. The temperature of the high-temperature exothermic peak shifts from 915 °C for W_45_Zr_40_Ti_7.5_Ni_7.5_ to 927 °C for W_55_Zr_30_Ti_7.5_Ni_7.5_ and further to 971 °C for W_65_Zr_20_Ti_7.5_Ni_7.5_. This systematic shift suggests that decreasing the Zr content delays the formation of the W-Zr intermetallic phase. A plausible explanation is that, with less available Zr and stronger competitive reactions between Zr and Ti/Ni at lower temperatures, the local chemical driving force for W_2_Zr formation is reduced. As a result, a higher temperature is required to initiate the W-Zr reaction.

**Figure 8:**
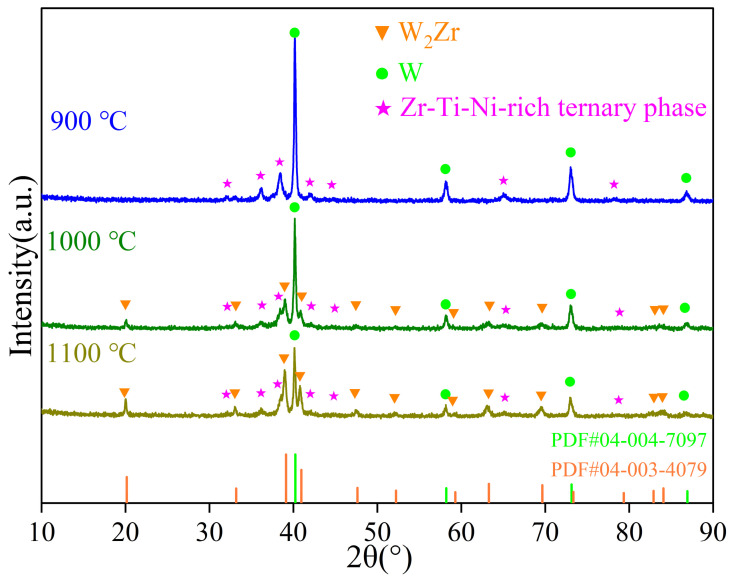
XRD patterns of the W_55_Zr_30_Ti_7.5_Ni_7.5_ alloys sintered at different temperatures.

**Figure 9:**
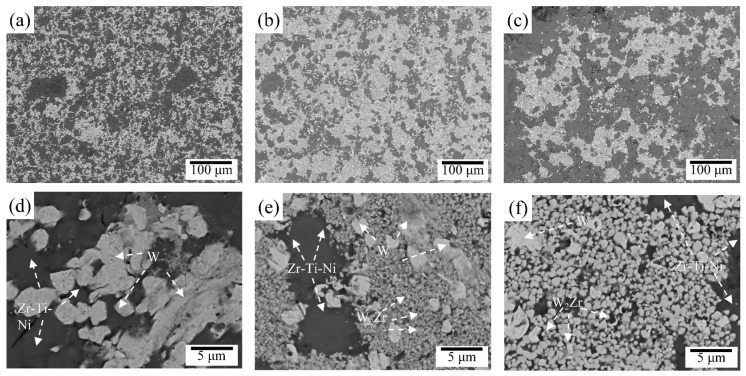
SEM images of the W_55_Zr_30_Ti_7.5_Ni_7.5_ alloy sintered at different temperatures: (**a**,**d**) 900 °C; (**b**,**e**) 1000 °C; and (**c**,**f**) 1100 °C.

Figure 8 and Figure 9 present the XRD patterns and SEM images of the W_55_Zr_30_Ti_7.5_Ni_7.5_ alloy sintered at different temperatures. The XRD pattern of the sample sintered at 900 °C shows no detectable diffraction peaks for the W_2_Zr intermetallic. Correspondingly, the SEM image (Figure 9d) reveals a microstructure of spherical W particles dispersed in a darker Zr-Ti-Ni matrix, indicating that Zr primarily reacts with Ti and Ni at this temperature, and the reaction between W and Zr does not occur. When the sintering temperature rises to 1000 °C, diffraction peaks of W_2_Zr appear, confirming the onset of the W-Zr reaction. However, because Zr is preferentially consumed by Ti and Ni during heating to form the Zr-Ti-Ni-rich ternary phase, the amount of free Zr available to react with W is substantially reduced. Consequently, despite the nucleation of W_2_Zr at this higher temperature, both its thermodynamic driving force and its growth space are limited, effectively suppressing its coarsening and excessive precipitation.

Based on a comprehensive analysis of the alloying process, it can be inferred that Zr preferentially reacts with Ti and Ni. According to the Johnson–Mehl–Avrami–Kolmogorov (JMAK) theory, the driving force for nucleation arises directly from the Gibbs free energy difference between the new and parent phases, and this Gibbs free energy is a function of the local compositional concentrations of the constituent elements. In other words, because the majority of Zr has already been consumed in reactions with Ti and Ni at lower temperatures, the atomic concentration of Zr in the vicinity of W is substantially reduced by the time the system reaches the temperature range where the W-Zr reaction would normally occur. This depletion of local Zr effectively suppresses the nucleation of W_2_Zr.

### 3.3. Dynamic Response Characteristics

**Figure 10:**
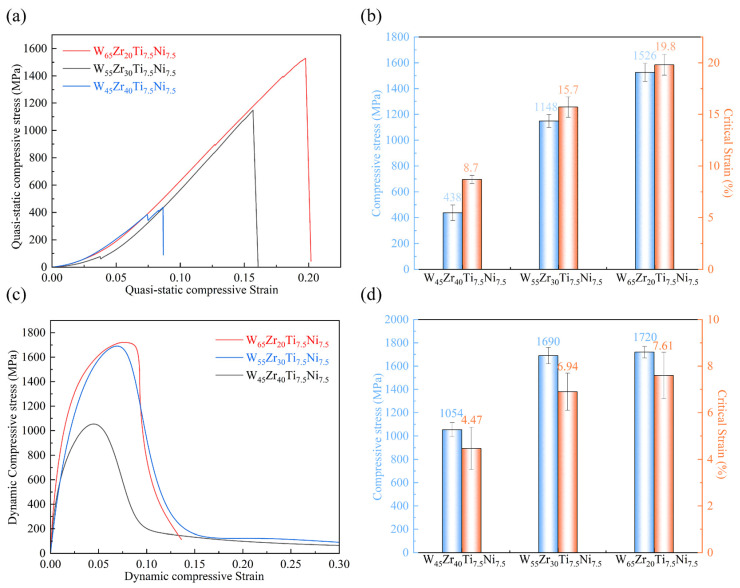
Stress–strain curves of W*_x_*Zr_85−*x*_Ti_7.5_Ni_7.5_ alloys: (**a**,**b**) quasi-static compression and (**c**,**d**) dynamic compression (8000 s^−1^).

To evaluate the effect of microstructure on the compressive response of the WZrTiNi alloys, quasi-static compression and split Hopkinson pressure bar (SHPB) tests were performed on the W*_x_*Zr_85−*x*_Ti_7.5_Ni_7.5_ specimens. Figure 10a shows that both the compressive strength and strain increase as the characteristic size of W_2_Zr decreases from the micrometer scale to the nanometer scale. The W_45_Zr_40_Ti_7.5_Ni_7.5_ alloy, which contains abundant coarse W_2_Zr particles, exhibits the lowest quasi-static compressive strength and strain (438 MPa and 8.7%, respectively). Under dynamic loading, its compressive strength increases to 1054 MPa because of strain-rate strengthening. However, the dynamic critical strain is only 4.47%, indicating a typical brittle feature.

As Zr content declines, the proportion of coarse W_2_Zr drops significantly. Meanwhile, more unreacted W is reserved, and the intermetallic phases are greatly refined. Such microstructural evolution relieves the local stress concentration induced by coarse brittle particles, which well accounts for the enhanced compressive properties in experiments. Among the investigated alloys, W_65_Zr_20_Ti_7.5_Ni_7.5_ exhibits the best compressive performance, with a quasi-static compressive strength of 1526 MPa and a compressive strain of 19.8%. Under dynamic loading, the compressive strength further increases to 1720 MPa, while the dynamic critical strain remains at 7.6%. This value is remarkably higher than that of alloys with high Zr content.

**Figure 11:**
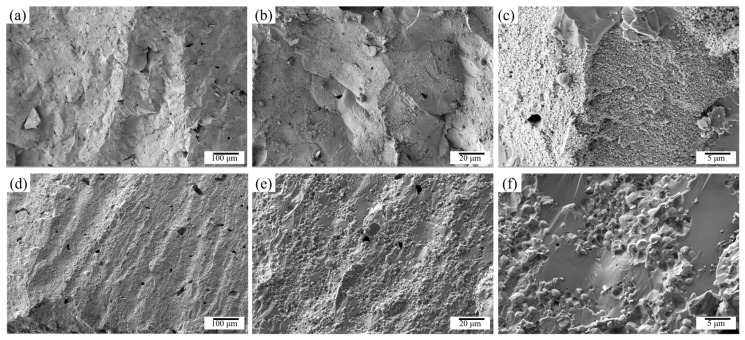
Fracture surface morphology after compression: (**a**–**c**) W_45_Zr_40_Ti_7.5_Ni_7.5_ and (**d**–**f**) W_65_Zr_20_Ti_7.5_Ni_7.5_.

Fracture surface analysis reveals that the W_45_Zr_40_Ti_7.5_Ni_7.5_ alloy (Figure 11a–c) exhibits typical brittle fracture characteristics, with cracks preferentially propagating along the interfaces between coarse W_2_Zr particles and the tungsten skeleton, indicative of pronounced intergranular fractures. In contrast, the W_65_Zr_20_Ti_7.5_Ni_7.5_ alloy (Figure 11d–f) features a significantly refined and uniformly distributed W_2_Zr phase, resulting in a fracture mode dominated by transgranular cracking and localized shear, with diminished intergranular separation. These observations confirm that reducing the Zr content effectively suppresses the coarsening of the brittle W_2_Zr phase, thereby optimizing the failure behavior under dynamic compression.

**Figure 12:**
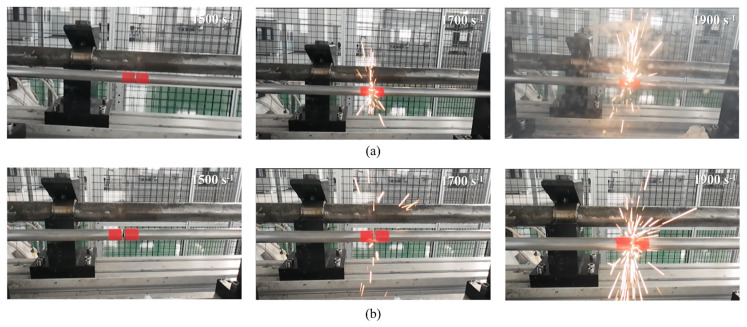
Energy-release photographs of the impact-induced reaction: (**a**) W_45_Zr_40_Ti_7.5_Ni_7.5_ and (**b**) W_65_Zr_20_Ti_7.5_Ni_7.5_.

Figure 12 compares the impact-induced reaction behavior of two representative alloys. Although the Zr content in W_65_Zr_20_Ti_7.5_Ni_7.5_ is only half that in W_45_Zr_40_Ti_7.5_Ni_7.5_, the luminous intensity and flame coverage observed during impact remain pronounced. This result indicates that the lower-Zr-content alloy can still maintain substantial impact-induced reactivity. A possible explanation is that the improved dynamic compressive response allows more strain energy to be stored before fracture, which may favor more extensive fragmentation and thus increase the exposed reactive surface area during impact. However, this interpretation should be regarded as a plausible explanation rather than direct proof, and further post-impact microstructural evidence would be required for verification.

In W-Zr-reactive structural alloys, Zr serves as the key reactive element responsible for impact-induced chemical reactions and energy release. Conventional wisdom holds that higher Zr content leads to greater energy-release capability in alloys when subjected to a high-velocity impact or detonation loading. Conversely, reducing the Zr content typically results in a significant decline in fragment reactivity and energy-release efficiency. The underlying mechanism of this anomalous behavior can be attributed to the synergistic regulation of deformation-induced energy storage and fragmentation characteristics through microstructural optimization. Brittle materials accumulate substantial strain energy during compression, which increases with the extent of elastic deformation. When brittle materials fracture from a state of low stored strain energy, they tend to fail by axial splitting. In contrast, when fracture occurs from a higher strain energy state, the failure mode transitions from splitting to expansive fragmentation, which dramatically increases the contact area between reactive constituents and the surrounding environment, thereby enhancing energy release.

## 4. Conclusions

(1) Leveraging the low-melting-point effect induced by multi-element alloying, the addition of Ti and Ni effectively reduces the sintering difficulty of W-Zr alloys. Experimental results show that Zr preferentially undergoes an exothermic reaction with Ti and Ni at approximately 760 °C to form the Zr-Ti-Ni-rich ternary phase, thereby consuming a large fraction of free Zr. Only when the temperature exceeds 900 °C does the residual Zr react with W to form the brittle W_2_Zr phase.
(2) By introducing Ti and Ni as binder-phase elements and tailoring the Zr content, effective control over the morphology and distribution of the brittle W_2_Zr phase in W-Zr alloys can be successfully achieved. With decreasing Zr content, the dominant phase in the alloy transitions from coarse, micrometer-scale W_2_Zr to elemental W, while the residual W_2_Zr phase is significantly refined to the nanoscale. This microstructural evolution suppresses stress concentration, delays crack initiation, and consequently enhances the mechanical strength of the material.
(3) The W*_x_*Zr_85−*x*_Ti_7.5_Ni_7.5_ alloy exhibits excellent combinations of mechanical properties and dynamic response characteristics. Notably, the W_65_Zr_20_Ti_7.5_Ni_7.5_ alloy exhibits a quasi-static compressive strength of 1526 MPa and a dynamic compressive strength of 1720 MPa. Its reaction threshold strain rate is approximately 1700 s^−1^, and the luminous intensity increases markedly with rising strain rate, indicating that this composition possesses excellent impact-induced energy-release potential while maintaining high load-bearing capacity.

## Figures and Tables

**Table 1:** Compositional details of different W*_x_*Zr_85−*x*_Ti_7.5_Ni_7.5_ alloys.

Alloy System	Theoretical Density (g·cm^−3^)	Composition (wt%)/(at%)			
		W	Zr	Ti	Ni
W_45_Zr_40_Ti_7.5_Ni_7.5_	9.11	45/25.3	40/45.3	7.5/16.2	7.5/13.2
W_55_Zr_30_Ti_7.5_Ni_7.5_	10.04	55/32.8	30/36.0	7.5/17.2	7.5/14.0
W_65_Zr_20_Ti_7.5_Ni_7.5_	11.17	65/41.3	20/25.6	7.5/18.3	7.5/14.9

**Table 2:** Experimental equipment.

Equipment	Model
Electronic balance	DLX-48 (Mettler Toledo, Greifensee, Switzerland)
Vacuum tantalum heat treatment furnace	HVF4060 (Zettl GmbH & Co. KG, Berlin, Germany)
Metal powder mixer	WD166 (Hosokawa Micron Corporation, Osaka, Japan)
Metallographic microscope	Axio Observer 7 (Carl Zeiss AG, Oberkochen, Germany)
X-ray diffractometer	Smart Lab (Rigaku Corporation, Akishima, Tokyo, Japan)
Scanning electron microscope	JSM-7900F (JEOL Ltd., Tokyo, Japan)
Transmission Electron Microscopy	Thermo Fisher Scientific Talos F200X G2 (Thermo Fisher Scientific Inc., Hillsboro, OR, USA)
Energy-dispersive X-ray spectrometer	Oxford X-Max 5 (Oxford Instruments NanoAnalysis, High Wycombe, UK)
Thermal analyzer	STA44 F3 (NETZSCH-Gerätebau GmbH, Selb, Germany)
Universal testing machine	Instron 5982 (Instron Corporation, Norwood, MA, USA)

**Table 3:** EDS date of WZrTiNi alloys.

Region	Content (wt%)/(at%)			
	W	Zr	Ti	Ni
A	78.27/61.82	18.82/29.96	1.83/5.55	1.08/2.67
B	9.16/3.71	52.54/42.95	16.27/25.34	22.04/28.00

## Data Availability

The original contributions presented in this study are included in the article. Further inquiries can be directed to the corresponding author.
